# Consumption and the South

**Published:** 1854-01

**Authors:** 


With this case, given as a precaution against the danger which attends taking daily out-door exercise, without medical advice, for any of the prominent symptoms of consumption, such as cough, short breath, quick pulse, debility, pains about the chest, &c., I here give, as being highly instructive, an article from "The Home Journal" of December 10th, 1853, under the editorial head:—

(*For Invalids only.*)

Are you quite well, dear reader? Are all those who are dear to you quite well? If so, perhaps you will kindly pass on to another topic, allowing me, under the Idlewild caption, for this week, to answer a letter from an invalid—the information, thus called for, being interesting to invalids only, or to those with precious invalids for whom they feel and care. In a world where mortals walk beside Death with a face averted, the sick can talk safely of their sorrows only to the sick. I do not claim, therefore, the attention due to a general topic. Though, with pulmonary consumption for our country's most fatal liability, any experience, in eluding or defeating it, may be of interest to so many, as to be, at least, excusably tedious to the remainder. It comes appropriately from Idlewild. The Highlands around us, I fully believe, are the nearest spot to New York, where the acrid irritation of our eastern and seaboard climate is unfelt. Poke your fire, then, dear delicate reader!—(for you are an invalid, by your following me thus far)—and settle yourself comfortably in your arm-chair, while I lay before you a sad and well-written letter from an invalid:

"*C*******, *November* 21, 1853.

"Mr. Willis.—*Dear Sir:* You will perhaps think it presumption in me, an entire stranger, to address you as I now do; but I shall be willing to abide your judgment after you have heard my story. I am a Presbyterian clergyman, in feeble health. After five years' preaching in one happy parish, my lungs gave out, and I was obliged to give up my calling. By the advice of physicians here and in New York, I spent two winters at the South, roaming from place to place, but spending most of the time in Jacksonville and St. Augustine, Florida. I was there during the winter of your tour in that region, and on the same sad errand. And I may here say, that I have taken great pleasure in reading, weekly, your record of travel in those parts.

"But I got no essential benefit from the 'Sunny South'—nothing but some disgust for it, weariness of travel, and a warmer love for the North and for my home. Neglecting further medical advice, I bought, two years since, a pleasant site for a country residence in this, my native place, built a house, and devoted myself to tree-planting and gardening of all sorts. This has been my sole employment for two summers. In winter I warm my whole house *moderately*, not allowing the mercury to rise above sixty or sixty-two degrees, and connect with this a thorough ventilation. I remain within doors most of the time. Between romping with my two children, playing with grace-sticks, battledoor, etc., fighting imaginary foes with my cane, and the music of a piano, I manage to get regular, daily exercise and recreation. In favorable weather I also take a brisk walk of half a mile.

"This mode of life makes me quite happy, and I enjoy a tolerable degree of health; but *I don't get well.* I followed you to Idlewild with much interest, having a fellow-feeling on one point, at least, and watched to see whether you would get the mastery of disease. In your last letter you say that you are no longer to be classed among consumptives. Alas! I can't say as much for myself, I fear. And on reading your lines, I resolved to write to you, as a once fellow-invalid, and ask, *What has cured you?* The doctors advise me to go South and take cod-liver oil, but their prescriptions do me no good; and I improve most when following my own judgment. I spade, and hoe, and rake quite lustily, and ride horseback, in summer; I cough but little, and eat and sleep as well as ever—but cannot use my lungs. Now, may I trouble you to give me some plain advice—a little of your own daily regimen—if you are willing to do so, an account of what has helped you.

"I consult you, not as a doctor, but as a man of benevolence, knowing by experience the feelings of a young man arrested by disease, and laid aside from the activities of life.

"If you do not think proper, or find it convenient, to address me personally, I beg leave to suggest that you give your friends, through the *Home Journal*, some of your views and your experience relating to the treatment of pulmonary affections. A

large and eagerly attentive audience would listen to your words, I assure you.

"Pardon me, sir, if I have annoyed you by this letter; and if you are willing to do so, please allow me to hear from you, and greatly oblige, yours, with true respect, A. D. G."

[To which straightforward and touching letter, the following was the bulk of my reply—not very satisfactory, I fear, though possibly there may be a point or so in which it is either suggestive or corroborative :]—

* * The politicians teach us how to treat a disease, I think. They do not try to *convert* the opposing party. They are content if they can *keep it in the minority*—sure that it will tire, in time, of its want of power, change sides, or disappear. The patient who troubles himself least about his disease, (or leaves it entirely to his doctor,) but who perseveringly *outvotes it* by the high condition of the *other parts* of his system, is the likeliest to recover—and it is of this *high condition*, alone, that I have anything to say. Of twenty who may be sleepless with a cough and weakened with the raising of blood, no two, perhaps, are subjects for precisely the same medical treatment, or diseased in precisely the same locality, though all are called "consumptives." Our friends, the physicians, are better geographers than we, as to where the healing is wanted, though they strangely confine themselves to the specific ailment, taking it for granted that the patient keeps the rest of his body in proper training for recovery. It is medical etiquette, I believe, to refrain from any very particular inquiry into this. But few sick men are wise or firm-minded enough to be safely trusted with their own general condition; and I, for one, came very near dying—not of my disease, but of what my doctors took for granted.

To leave generalities, however, and come to the personal experience which you ask for:

I went to the Tropics, as a last hope, to cure a chronic cough and blood-raising which had brought me to the borders of the grave. I found a climate in which it is hard to be unhappy about anything—charming to live at all—easy to die. (At least, those who were sure of dying, and did die—and in whose inseparable company I thought I was—were social and joyous to the last.) The atmosphere of that Eden-latitude, however, is but a pain-stilling opiate, while the Equator might be called a kitchen-range for a Sardanapalus, and the Antilles are but tables loaded with luxuries. The Caribbean Sea is the Kingdom of the Present Moment. The Past and the Future are its Arctic and Antarctic —unthought of, except by desperate explorers. Hither are sent invalids, with weakened resolution, to make a pilgrimage with prescription and prudence! You may see by the book I have just published, (Health-Trip to the Tropics,) with what complete forgetfulness of care or caution I made one of an invalid company for months. Was anybody going to be shut up in a bed-room with such nights out of doors? Was anybody going to be dull and abstinent with such merry people, and a French break-fast or tempting dinner on the table?

I reached home in July, thoroughly prostrated, and, in the opinion of one or two physicians, a hopeless case. Coughing almost the whole of every night, and raising blood as fast as my system could make it, I had no rest and no strength. I lingered through the summer, and, as the autumn came on, and the winter was to be faced, I sat down and took a fair look at the probabilities. With the details of this troubled council of war, I will not detain you; but, after an unflinching self-examination, I came to the conclusion that I was, myself, the careless and indolent neutralizer of the medicines which had failed to cure me—that one wrong morsel of food or one day's partially-neglected exercise might put back a week's healing—and that, by slight omissions of attention, occasional breaking of regimen, and much too effeminate habits, I was untrue to the trust which Gray, my friend and physician, had made the ground of his prescriptions. And, to a minutely persevering change in these comparative trifles, I owe, I believe, my restoration to health. There was not a day of the succeeding winter, however cold or wet, in which I did not ride, eight or ten miles, on horseback. With five or six men, I was, for most of the remaining hours of the day, out of doors, laboring at the roads and clearings of my present home. The cottage of Idlewild was then unbuilt, and the neighboring farm-house, where we boarded, was, of course, indifferently warmed; but, by suffering no state of the thermometer to interrupt the morning cold bath, and the previous friction with flesh-brushes, which makes the water as agreeable as in summer, I soon became comparatively independent of the temperature *in* doors, as my horse and axe made me independent of it when *out* of doors. With proper clothing to resist cold or wet, I found (to my surprise) that there was no such thing as disagreeable weather to be felt in the saddle; and, when a drive in a wagon or carriage would have intolerably irritated my cough, I could be all day in the woods with an axe, my lungs as quiet as a child's.

**To be continued.**

